# Comparative transcriptome analyses reveal insights into catkin bloom patterns in pecan protogynous and protandrous cultivars

**DOI:** 10.1371/journal.pone.0281805

**Published:** 2023-02-16

**Authors:** Hormat Shadgou Rhein, Avinash Sreedasyam, Peter Cooke, Ciro Velasco-Cruz, Jane Grimwood, Jeremy Schmutz, Jerry Jenkins, Sajal Kumar, Mingzhou Song, Richard J. Heerema, L. J. Grauke, Jennifer J. Randall

**Affiliations:** 1 Molecular Biology Program, New Mexico State University, Las Cruces, New Mexico, United States of America; 2 HudsonAlpha Institute for Biotechnology, Huntsville, Alabama, United States of America; 3 Microscopy Core Facility, New Mexico State University, Las Cruces, New Mexico, United States of America; 4 Department of Entomology, Plant Pathology, and Weed Science, New Mexico State University, Las Cruces, New Mexico, United States of America; 5 Department of Computer Science, New Mexico State University, Las Cruces, New Mexico, United States of America; 6 Departments of Plant and Environmental Sciences and Extension Plant Sciences, Las Cruces, New Mexico, United States of America; 7 USDA ARS Pecan Breeding and Genetics, Somerville, Texas, United States of America; Universidad Politecnica de Cartagena, SPAIN

## Abstract

In perennial plants such as pecan, once reproductive maturity is attained, there are genetic switches that are regulated and required for flower development year after year. Pecan trees are heterodichogamous with both pistillate and staminate flowers produced on the same tree. Therefore, defining genes exclusively responsible for pistillate inflorescence and staminate inflorescence (catkin) initiation is challenging at best. To understand these genetic switches and their timing, this study analyzed catkin bloom and gene expression of lateral buds collected from a protogynous (Wichita) and a protandrous (Western) pecan cultivar in summer, autumn and spring. Our data showed that pistillate flowers in the current season on the same shoot negatively impacted catkin production on the protogynous ‘Wichita’ cultivar. Whereas fruit production the previous year on ‘Wichita’ had a positive effect on catkin production on the same shoot the following year. However, fruiting the previous year nor current year pistillate flower production had no significant effect on catkin production on ‘Western’ (protandrous cultivar) cultivar. The RNA-Seq results present more significant differences between the fruiting and non-fruiting shoots of the ‘Wichita’ cultivar compared to the ‘Western’ cultivar, revealing the genetic signals likely responsible for catkin production. Our data presented here, indicates the genes showing expression for the initiation of both types of flowers the season before bloom.

## Introduction

Pecan (*Carya illinoinensis*) trees are monoecious, forming staminate and pistillate flowers on separate inflorescences. Pollination in pecan is anemophilous (wind pollinated) and trees manifest dichogamy, meaning that staminate and pistillate flowers of an individual genotype are functional at different times [1]. In protandrous pecan trees, staminate flowers mature and shed their pollen prior to pistillate flower receptivity, while in protogynous trees, pistillate flower maturation and receptivity occur prior to pollen shed [2]. This flowering feature favors outcrossing and contributes to the typically high level of heterozygosity of individuals in this species [2]. Separation in male and female bloom periods may be complete or incomplete, granting some overlap between pollen shed period and pistillate receptivity within some genotypes and, thus, the opportunity for limited self-pollination [2].

Pecan staminate inflorescences consist of two to four groups of catkins or aments borne from lateral buds on shoots from the previous season growth. Each group usually contains three or more individual catkins that are joined to a common stalk. Development of staminate inflorescences occurs in two phases. The first phase for the development of staminate inflorescence is ‘initiation’ which begins the summer prior to bloom the following year [3]. The initiation of catkin primordia occurs about two weeks after active growth [3]. During the ‘initiation’ phase specific genes begin to be expressed, which primes for the development of staminate inflorescence prior to the second phase where tissue differentiation occurs and morphological changes can be observed. In protandrous cultivars such as ‘Western’ morphological changes and full staminate differentiation occur in the first season prior to the winter. In protogynous cultivars such as ‘Wichita’ the development of the staminate inflorescence is paused in the winter and the completion of differentiation happens the following season after the bud break [3]. The variation in catkin primordia differentiation between protandrous and protogynous trees can only be distinguished after the first summer season. In both types, bract differentiation occurs about 10 days before anthers [4]. Protandrous cultivars differentiate their inflorescence initials in the spring and summer of the first season and anther primordia differentiates about 3 to 4 months after bud break, anthers become bilobed in the subsequent spring following bud break. In protogynous genotypes, catkin primordia and bracts differentiate at the same time as protandrous cultivars, but the initiation of the floral apex, anther primordia, stamen primordia and the bilobed anther do not occur until the next spring [5, 6].

Pecan shoots that mature a fruit cluster in a given year are less likely to bear fruit the subsequent year and non-fruiting shoots in that same given year are more likely to bear fruit in the subsequent year, indicating that alternate bearing occurs in pecan at a shoot-to-shoot level [7, 8]. Several studies have reported transcriptome data on pecan trees and gene expression of flower specific tissues in pecan [9–12]. However, the expression of genes involved in individual flower initiation and the patterns of staminate inflorescence on fruiting and non-fruiting shoots have not been previously reported. Here we present staminate inflorescence patterns and the impact that the presence of current pistillate flowers have on catkin numbers on the same shoot and the impact of the shoot bearing fruit the year before on catkin numbers on the same shoot the next season. To identify potential genes involved in staminate inflorescence initiation/differentiation and the timing of their expression, we explored correlations between staminate inflorescence production and gene expression of buds collected at different time points in the summer and spring from a protogynous (‘Wichita’) cultivar and protandrous (‘Western’) cultivar.

## Materials and methods

### Staminate bloom

Three mature ‘Western’ and three mature ‘Wichita’ trees at the New Mexico State University Leyendecker Plant Science Center (Lat. 32˚198636 N, long. 106˚741284 W; elevation 1176 m) were selected for staminate bloom data collection. The genotypes of the trees were confirmed through genotyping using the primers developed by Grauke et al., 2003 (Table 1). In the spring of 2019, from each tree, 40 shoots were selected (20 shoots producing and 20 shoots with no pistillate flower at the time of the data collection). The number of individual catkins were counted on each shoot. The fruiting or non-fruiting status of every individual shoot for the previous year was also recorded by the presence of a dried rachis or rachis scar.

**Table 1:** Base sequence and characteristics of the two primers used for genotyping the pecan trees [13].

Primer Name	GeneBank Accession	Primer Sequence	Repeat Motif	Expected Size	Annealing Temp (°C)
PM-CIN4	AY218227	GGCATCAGAGAAGGCTCCT (forward) CTCACCCGTCTCTAGGGCTA (reverse)	(CTT)2, (CTT)12	112	57
PM-CIN13	AY218229	CCGCAGATGGTTTGAAGAA (forward) ACAAATTCCTCACTCCGGAG (reverse)	(GAA)14,(GAT)2	117	54

### Bud microscopy

To see the morphological differences in formation of catkin primordia in bud tissues, 15 dormant buds from ‘Western’ and 15 dormant buds from ‘Wichita’ cultivars collected in February were excised. Samples were fixed in 2.5% glutaraldehyde solution buffered with 0.1M imidazole-HCl and stored at 4°C. Vertical dissection of individual dormant buds was performed using a clean, stainless steel razor blade into two halves, samples were placed on sands under water and were first examined using a model M165FC stereofluorescence microscope (Leica Microsystems, Buffalo Grove, IL) using the GFP1 and Violet filter sets to evaluate favorable planes through the presumptive tissues. Then, for Laser scanning confocal microscopy, the cut surfaces of the selected buds were mounted on the coverslip surface of glass-bottom microwell dishes (MatTek Corp., Ashland, MA) and examined with a model TCS SP5II confocal microscope system (Leica Microsystems, Exton, PA) using the 488 nm Argon laser line for fluorescence excitation and a 20x long working distance objective lens. Fluorescence emission was collected in three channels, green from 500–570 nm, yellow-orange from 580–620 and red from 650–720 nm, as stacks of optical sections approximately 30–40 micrometers deep. Final images of comparable buds were displayed as graphic overlays of the three channels.

### Plant material for RNA-seq

Bud tissue samples from two pecan cultivars, ‘Wichita’ and ‘Western’, were collected in June 13 June 21, 2017, and September 16, 2016, and March 5, and March 22, 2018. Samples were collected from three individual trees for each cultivar from the same location as samples collected for microscopy. On each tree, four fruiting shoots and four non-fruiting shoots were randomly selected for sample collection. The lateral buds were removed and frozen directly into liquid nitrogen. For each tree, bud samples from fruiting shoots were bulked together and samples from non-fruiting shoots of each tree were bulked together. Samples from different trees kept separated and used as replicates. In March, there was not yet any active growth or pistillate flowers formed, so the fruiting/non-fruiting samples were labeled based on the shoot fruiting status from the previous year (i.e., shoots were categorized into those that produced fruit (fruiting) and those that produced no fruit (non-fruiting) the previous season). Samples were homogenized separately with mortar and pestle using liquid nitrogen. Approximately 100 mg of frozen tissue was utilized for RNA extraction. Total RNA was extracted using plant/fungi total RNA purification kit (Norgen Biotek, Ontario, Canada) according to manufacturer`s instruction. All samples were DNase treated using the Qiagen DNAse kit to remove any DNA contamination. Sequencing libraries were constructed using an Illumina TruSeq stranded mRNA library kit (20020595) and TruSeq RNA UD Indexes (20022371) using standard protocols. Libraries were sequenced on a NovaSeq 6000 using paired ends and a read length of 150 base pairs.

### RNA-seq analyses

All reads were trimmed for adapter, quality, and length. Reads were trimmed using quality scores with 0.05 limit through automatic and/or trim adapter list to remove the read-through adapters. Then all the reads were trimmed to remove a maximum of 2 ambiguous bases at either 3`or 5`end of the reads and reads shorter than 5 nucleotides length were discarded. Trimmed reads were then mapped to the pecan reference genome of ‘87MX 3.2–11’ V.1.1 (Phytozome). Read alignment performed with a mismatch cost of 2, insertion and deletion costs of 3, length and similarity fraction of 0.8 and a maximum number of 10 hits for a read. Differential gene expression analyses were performed using CLC Genomics workbench 12.0.2 (https://digitalinsights.qiagen.com). All transcriptomes were analyzed for a selection of 1229 genes due to their known function in flowering in other species (*Arabidopsis thaliana* and *Juglans regia*) in addition to highly expressed genes in pecan ‘bud and catkin’ and ‘bud and pistillate’ tissue specific analyses. All the analyses were based on a log2 fold change of higher than 1.5 and adjusted p-value of less than 0.05. The RNA-Seq data reads are available in NCBI BioProject PRJNA782058.

### Staminate bloom patterns and statistical analyses

A Poisson regression analysis was carried out to study the catkin bloom patterns. The link function was the square root function. The regression model includes the fruiting and non-fruiting, and variety as independent variables, and the number of catkins as the response. In order to correct for the heteroscedasticity due to the shoots, an issue detected by the residual analysis, the model includes a variance component for each shoot. The numerical analysis was performed with Proc Glimmix, SAS 9.4, with the statement random _resid_ /group = shoot.

### qRT-PCR

RNA was extracted from the same tissues using the method explained above. Extracted RNA were DNAse treated to remove any contaminant DNA. For each sample, 15 μl of RNA was mixed by 2 μl of 10X DNase Buffer, 1 μl of DNase I and 1μl of sterile water according to the manufacturer protocol (Deoxyribonuclease I, Amplification Grade, Invitrogen, Grand Island, NY). The reaction tube was incubated at room temperature for 15 min, then 1 μl of 25 mM EDTA solution was added to each reaction followed by the second incubation at 65°C for 10 min. The DNase-treated RNA was quantified using a NanoDrop 1000 spectrophotometer (Thermo Scientific, Wilmington, DE) and was stored at -80°C freezer. 20 ng of DNase-treated RNA was utilized for synthesizing the complementary DNA (cDNA) using a SuperScript® IV Reverse Transcriptase kit (Invitrogen, Carlsbad, CA). For the initial reaction 1 μl of 50 μM oligo dT primer, and 1.0 μl of 10 mM dNTP mix was added to 20 ng DNAse-treated RNA. Sterile water was added to bring the volume to a total of 14 μl. Reaction was incubated at 65°C for 5 min followed by a one-minute incubation on ice and vortexed for 3 seconds. Then, 4 μl of 5X SuperScript™ IV buffer, 1 μl of SuperScript™ IV Reverse Transcriptase and 1 μl of 100 mM DTT were added to the reaction tube based on the manufacturer protocol (Life Technologies, Grand Island, NY). The reaction tubes (total volume of 20 μl) were then incubated at 52.5°C for 10 minutes followed by another 10-minute incubation at 80°C. cDNA was stored at -20°C for qPCR assays. Gene specific primers and probes were designed for *GI*, *MS2*, *STM*, *KNAT6*, *TFL1* and *CAL-A* (Table 2) and synthesized by Integrated DNA Technologies (Coralville, IA) each with a unique fluorescent reporter dye. The *ACTIN* probe [14] was used as the housekeeping gene to normalize the qPCR analyses. Assays for each sample were performed in 3 technical and 3 biological replicates using iQ™ Multiplex Powermix (Bio-Rad). Quantitative PCR wells for each sample consisted of 20 ng (1 ul) of cDNA, 1X concentration of each probe, and sterile water into a 10 μl final volume. Pecan genomic DNA was used as a positive control, and at least three negative controls were included in each reaction plate. The qPCR was performed using a CFX96 touch real-time detection system (BioRad, Hercules, CA). The qPCR protocol was performed as explained by Thompson et al., 2021 for 95°C for 2.5 min, followed by 39 cycles of 95°C for 15 s, and 60°C for 60 seconds. BioRad software of the CFX Touch Real-Time PCR Detection System measured Starting quantities (SQ) based on standard curves of genomic DNA dilution series that were added to each plate in triplicate.

**Table 2:** The sequence of primers/probes used for qPCR analysis.

Target Gene	Assay	Sequence
*ACTIN*	Forward primer Probe Reverse Primer	TTGTATGTGGTCTCGTGGATTC /56-FAM/TGGAAGAGA/ZEN/ACTTCTGGGCAACGG/3IABkFQ/ ATCACAATTGGAGCTGAGAGG
*STM*	Forward primer Probe Reverse Primer	GAGGTAAAGGGAGGAGTAGAC /56-FAM/AGTGTCTGC/ZEN/ATTAGGATGATGATGACT/3IABkFQ/ CTATGGCTTATGTCCAGTGATG
*KNAT6*	Forward primer Probe Reverse Primer	GCCTTCCTAGCTACCTTTATC /5HEX/ACATCGCAG/ZEN/TTGTAGTCCAC/3IABkFQ/ CTTTCTCCTGTCCGTCTTTAAT
*MS2*	Forward primer Probe Reverse Primer	CATGACCACAGAGAAGCTG /56-FAM/AGTGGTGCA/ZEN/GAGGCAGATGTGTTT/3IABkFQ/ CGATGCACTTGGGATCAA
*GI*	Forward primer Probe Reverse Primer	GATGCAAGTGGGACAATGA /5Cy5/TTACACCAGGGCATCATCAC/3IAbRQSp/ GCACTGGCGCATGTATTA
*CAL-A*	Forward primer Probe Reverse Primer	CGATTAGACCACCACATCTTC /5Cy5/ACTAGGGTTTGGTTATGCAACTAG/31AbRQSp/ CTTGCTTCGAGATTCGGATAC
*TFL1*	Forward primer Probe Reverse Primer	GCTCATGCCTTCTGTCATT /56-FAM/AGTGTATAA/ZEN/GCAATCCTCATGTCGTCCC/3IABkFQ/ AGCATCCGGATCTGTCATA

## Results

### Catkin counts and statistical analysis for ‘Western’ and ‘Wichita’

**Fig 1:**
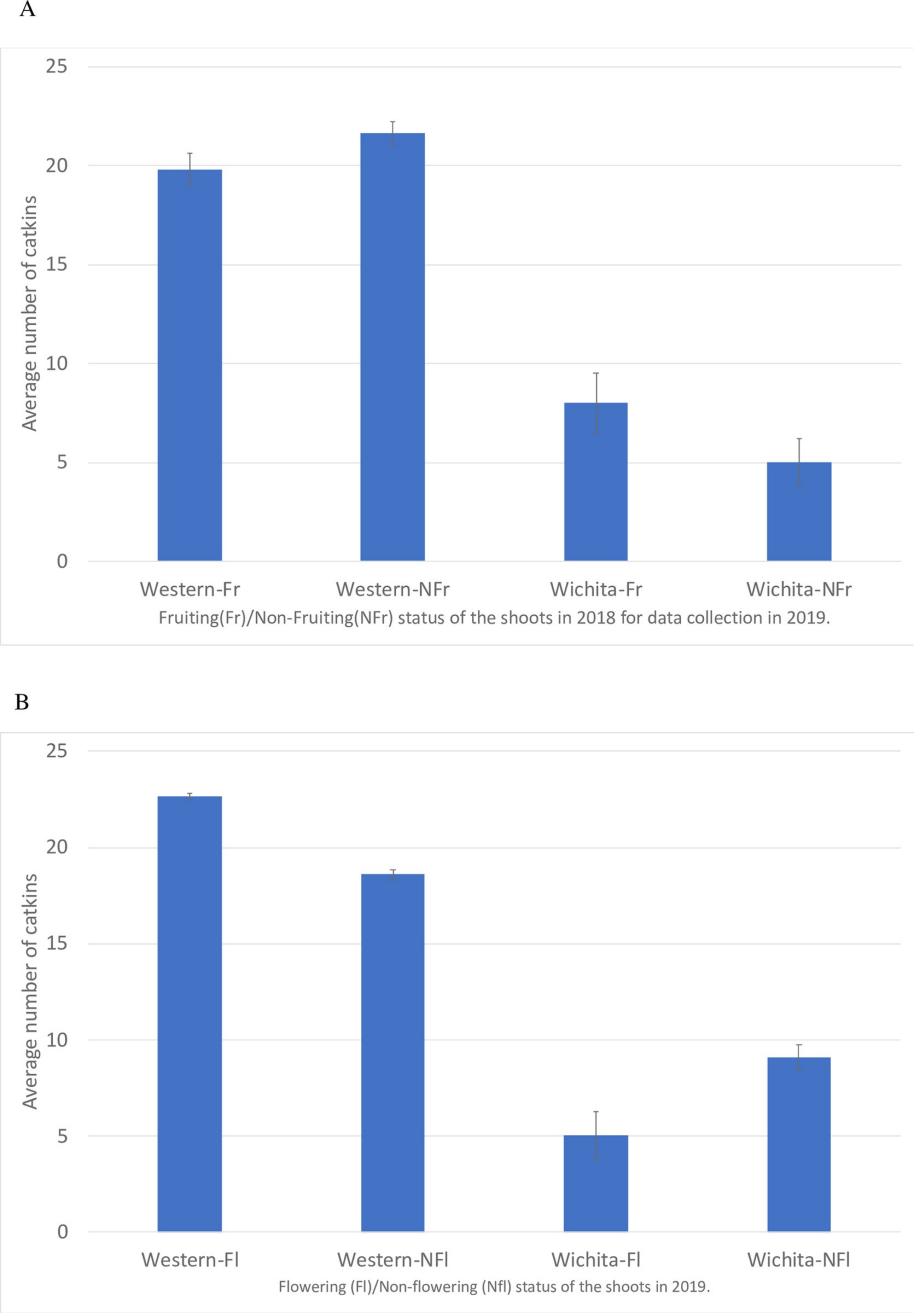
Catkin bloom patterns for ‘Western’ and ‘Wichita’. The number of catkins on a given ‘Wichita’ shoot was negatively correlated with the presence of pistillate flowers on the same shoot (p-value < 0.0001). The presence of fruit the previous year also had a positive impact, and a significant increase of catkins was observed than compared to shoots that were not fruiting the year before (p-value < 0.01 for the previous year). (A) This graph indicates the number of catkins on ‘Western’ and ‘Wichita’ on shoots that had produced fruit (Fr) the previous year and those shoots with no fruit production the previous year (NFr). In ‘Wichita’, the non-fruiting shoots in 2018 had fewer catkins produced on the same shoots in 2019. (B) This graph indicates the number of catkins on ‘Western’ and ‘Wichita’ on shoots that had pistillate flowers (Fl) during the current season year and those shoots with no pistillate flowers (NFl) during the current season. The ‘Wichita’ shoots with pistillate flowers had less catkins produced during the same year (2019) while no significant effect was observed on ‘Western’ catkin production.

To ascertain the impact of fruiting/non-fruiting on the production of catkin flowers the next year, we counted catkins from ‘Western’ and ‘Wichita’ trees. The previous year fruiting status, pistillate flower formation on the same year and the catkin counts were recorded and taken into consideration for catkin bloom analyses. The data from this study indicated that in 2019 there were an average of 5.0 individual catkins on ‘Wichita’ shoots that were non-fruiting in 2018 compared to the 8.2 individual catkins on ‘Wichita’ shoots that were fruiting in 2018 (Fig 1A). However, there were an average of 5.0 individual catkins on ‘Wichita’ shoots that had pistillate flowers during spring 2019 and 9.5 individual catkins on ‘Wichita’ shoots with no pistillate flowers during spring 2019 (Fig 1B). The number of catkins on a given ‘Wichita’ shoot was negatively correlated with the presence of pistillate flowers on the same shoot. In ‘Wichita’ the data indicates that there was a decrease in the number of catkins (p-value < 0.0001) when pistillate inflorescences were present (current year) on the same shoot. The presence of fruit the previous year also had a positive impact, and a significant increase of catkins was observed than compared to shoots that were not fruiting the year before (p-value < 0.01 for the previous year). However, ‘Western’ catkin production was not significantly impacted by previous fruit development or current pistillate production on the shoots (Fig 1A and 1B, S1 Table).

### Microscopy analysis confirms differences in anther development between protandrous and protogynous pecans

**Fig 2:**
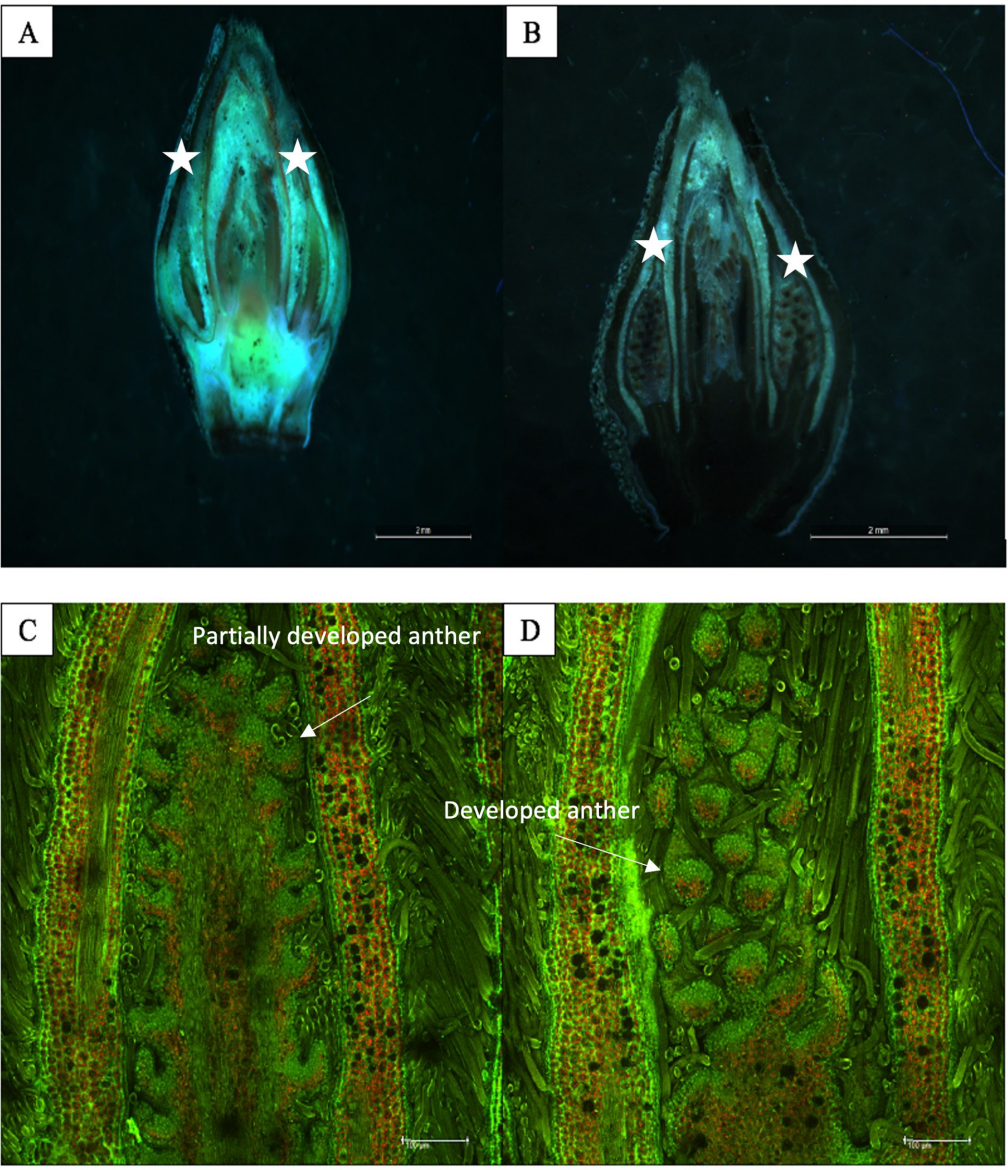
Microscopic images of the dormant buds of ‘Wichita’ and ‘Western’ pecan cultivars. Dormant buds were collected in February 2019. Buds were fixed with 2.5% glutaraldehyde and hand sectioned prior to confocal microscopy. (A) Overview of the entire ‘Wichita’ bud on a stereoscope. Catkin structures are observed on the right and left side of the bud (designated with a *). (B) Stereoscope overview of a ‘Western’ bud, more developed catkin structures are observed on the right and left side of the bud (designated with a *). (C) Confocal microscopy of partially formed catkin on ‘Wichita’ bud. (D) Confocal microscopy of fully formed catkin showing anthers on the ‘Western’ bud.

Catkins on protandrous cultivars such as ‘Western’, bloom and shed pollen generally earlier than protogynous cultivars such as ‘Wichita’. The microscopy images of the buds collected in February 2019, before the beginning of the growing season, indicate the differences in catkin primordia formation/differentiation between ‘Western’ and ‘Wichita’ that occurred the previous season. In Fig 2A and 2B, the stereoscope images show the main structure of the buds. The catkin structures are visualized on the right and left sides of the buds. Comparison of the stereoscope images displays advanced development of catkins in Fig 2B than 2A. The confocal microscope was used to image the same bud sections and specifically the catkin areas (Fig 2C and 2D). Comparison between Fig 2C and 2D clearly illustrates the structural differences between the catkin portion of the buds which are attributed to full anther development in ‘Western’ buds as compared to the ‘Wichita’ buds that did not have full anther development.

### RNA-seq analysis of the buds collected from pecan trees the season before bloom

Three genotyped ‘Western’ and three genotyped ‘Wichita’ pecan trees were used for collection of buds at different time points during the summer 2017 season. For each sample, four individual buds were collected and pulled. For the ‘Fruiting’ samples, buds were collected from shoots that were bearing fruit and for non-fruiting samples, buds were collected from shoots that were not bearing fruit. There was a total of 12 samples for each timepoint (three samples with four biological replicates/sample). For the 48 libraries represented in this study there were a total of 446,893,776 reads of which 96.4% were mapped to the 87Mx3-2.11 pecan genome [11]. All sequences have been deposited with NCBI BioProject PRJNA782058.

#### Fruiting vs. non-fruiting

**Fig 3:**
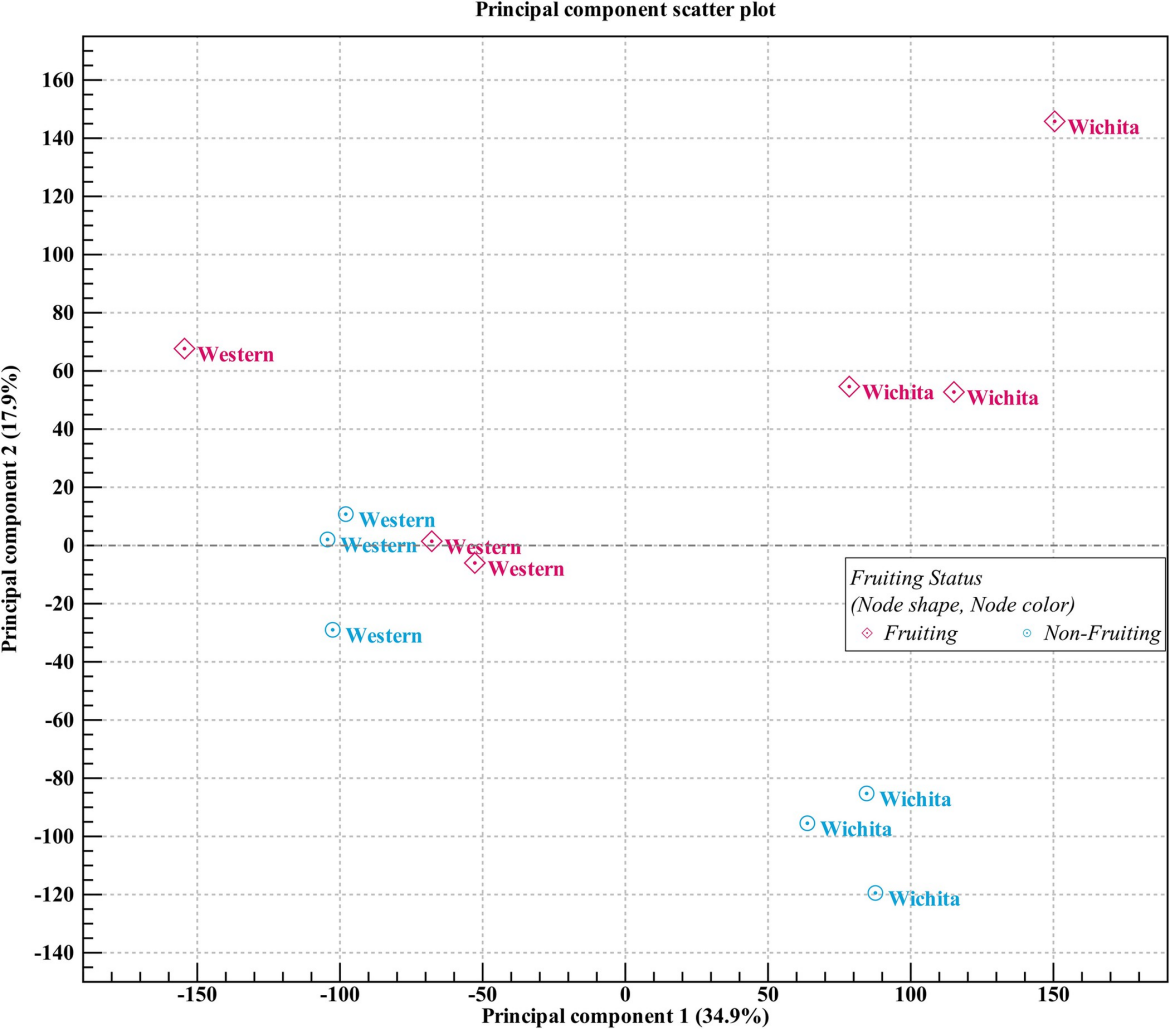
PCA plot of the transcriptomes of fruiting and non-fruiting samples of ‘Western’ and ‘Wichita’ cultivars. Samples were clustered separately in the PCA plot showing the first and second PCs which together explained 52.8% of variances. Fruiting and non-fruiting samples from Wichita clearly clustered separately revealing more differences between them in comparison to Western samples.

The principal component analysis (PCA) plot (Fig 3) and the Venn diagrams (S1 Fig) of the RNA-seq analyses of samples collected in September 2016 and June 2017, indicated a higher number of differentially expressed genes (DEGs) between the fruiting and non-fruiting samples from the ‘Wichita’ cultivar compared to the fruiting and non-fruiting samples from the ‘Western’ cultivar. As non-fruiting shoots of ‘Wichita’ most likely will produce more pistillate and catkin flowers the next year (compared to fruiting shoots) (Fig 1), some of the DEGs from the fruiting and non-fruiting ‘Wichita’ samples could be male specific genes. These data further emphasized the impact current pistillate production has on catkin production at the shoot level of the ‘Wichita’ cultivar in the same season while it had no significant effect on ‘Western’ (Fig 1).

In June 2017, there were only two DEGs between ‘Western’ non-fruiting and fruiting samples, *MALE STERILITY 2 (MS2)* and a non-characterized gene (S1A Fig). *MS2*, in some plant species, encodes a protein that is involved in male gametogenesis and is required for proper pollen development and in other plant species, is involved with male sterility [15, 16]. *MS2* expression was upregulated in ‘Western’ non-fruiting sample compared to fruiting sample in June 2017 and was upregulated in ‘Wichita’ in September 2016. Although *MS2* function in pecan is yet to be studied, the expression pattern of *MS2* in our study suggests the putative role of *MS2* in pecan catkin production.

In comparison, during the month of June 2017, there were 36 differentially expressed genes between ‘Wichita’ (protogynous) non-fruiting and fruiting samples (S1 Fig). These genes, listed in Table 3, include genes that have been described in other species as necessary for initiation of both pistillate and staminate flowers [15–20]. From these 36 genes, 10 genes were upregulated in ‘Wichita’ non-fruiting sample and 26 were upregulated in ‘Wichita’ fruiting sample. Further investigation of the function of these genes in future studies, could reveal substantial understanding of flower initiation in pecan. In September, there were 10 unique DEGs between ‘Western’ non-fruiting and fruiting samples and 108 unique DEGs between ‘Wichita’ non-fruiting and fruiting samples.

**Table 3:** Selected DEGs between non-fruiting (NFr) and fruiting (Fr) samples collected in June 2017 and September 2016 from ‘Wichita’(WI) and ‘Western’ (WE) cultivars (‘Wichita’ non-fruiting vs. ‘Wichita’ fruiting / ‘Western’ non-fruiting vs. ‘Western’ fruiting). The samples in second column had the higher expression of the genes. The cut-off for the DEGs were set to a minimum log2fold change of 1.5 with adjusted p-value of less than 0.05.

Collection time	Sample Type	Gene	GO/KEGG pathway
**June**	WE-NFr	*MS2*	Pollen development
**June**	WI-NFr	*SUF4*	Gamete fusion
**June**	WI-NFr	*UFO*	Floral meristem identity
**June**	WI-Fr	*RAV1*	Early flowering
**June**	WI-Fr	*RPK1*	Embryo development
**June**	WI-Fr	*WRKY19*	Flower initiation
**June**	WI-Fr	*WRKY75*	Flower initiation
**June**	WI-Fr	*LIR1*	Vegetative growth
**June**	WI-Fr	*MYB24*	Stamen development
**June**	WI-Fr	*MYB108*	Stamen development
**September**	WE-NFr	*ETR2*	Female flower formation
**September**	WE-NFr	*AMY1*	Flower repressor
**September**	WI-NFr	*JMJ30*	Flower repressor
**September**	WI-NFr	*MS2*	Pollen development
**September**	WI-NFr	*GASA14*	Plant growth
**September**	WI-NFr	*AIL1*	Downstream of *CO/FT*
**September**	WI-NFr	*ARR5*	Flower induction?
**September**	WI-NFr	*GRF7*	Pistillate development
**September**	WI-Fr	*ERF1B*	Subset of *AP2*
**September**	WI-Fr	*NFD4*	Female gametophyte development
**September**	WI-Fr	*NAC056*	Seed morphogenesis
**September**	WI-Fr	*PRR9*	*CO* activation
**September**	WI-Fr	*MYB108*	Stamen development
**September**	WI-Fr	*MARD1*	Seed dormancy
**September**	WI-Fr	*LSH4*	Suppression of organ differentiation
**September**	WI-Fr	*GSO1*	Embryogenesis

Out of the 10 DEGs between ‘Western’ non-fruiting and fruiting samples, two genes (*ETR2* and *AMY1*) were upregulated in ‘Western’ non-fruiting samples and the remainder were upregulated in the fruiting samples.

From the 108 unique DEGs between ‘Wichita’ non-fruiting and ‘Wichita’ fruiting samples in September 2016, 54 genes were upregulated in non-fruiting samples and 54 genes were upregulated in fruiting samples. *JMJ30*, *MS2*, *GASA14*, *AIL1*, *ARR5*, and *GRF7* (Table 3) were some of the upregulated genes in ‘Wichita’ non-fruiting samples. *ERF1B*, *NFD4*, *NAC056*, *APRR9*, *MYB108*, *MARD1*, *LSH4* and *GSO1* (Table 3) were some of the upregulated genes in fruiting samples.

S2 and S3 Figs indicate the log2 fold changes for the selected DEGs between ‘Wichita’ non-fruiting and fruiting samples in June 2017 and September of 2016. Many of these genes are known to specifically regulate flowering in other plant species. These genes included *SUF4*, *EC1*, *UFO*, *MYB*, *MYB 24*, and *MYB108* [17–22]. In addition to genes that were anticipated to have role in male flower production, genes with known function in female flower development [23, 24] were also differentially expressed in our samples collected in the season before bloom. *ETR2* was upregulated in ‘Western’ non-fruiting buds collected in September 2016. Also, *GRF7* and *NFD4* were upregulated in ‘Wichita’ samples collected from non-fruiting and fruiting shoots respectively. There were several DEGs among our samples that may play a role in initiation of both male and female flowers such as *AIL1*, *PRR9*, etc., (Table 3). The genes listed in this study are candidate genes for further investigation of their function in pecan flower initiation mechanisms.

#### ‘Western’ vs ‘Wichita’

Comparisons were also made between the ‘Wichita’ and ‘Western’ buds from June 2017 and September 2016. There were 107 DEGs between ‘Wichita’ and ‘Western’ non-fruiting samples. Forty-eight of these genes were specific and unique to the non-fruiting samples, and 16 DEGs were specifically upregulated in the ‘Wichita’ non-fruiting samples while the remainder were upregulated in ‘Western’ non-fruiting samples (S4 Fig) (Table 4).

**Table 4:** Selected DEGs between samples collected from ‘Western’ (WE) and ‘Wichita’ (WI) in June 2017 and September 2016 (‘Wichita’ fruiting (Fr) vs. ‘Western’ fruiting / ‘Wichita’ non-fruiting (NFr) vs. ‘Western’ non-fruiting). Second column is the sample that had higher expression. The cut-off for the DEGs were set to a minimum log2fold change of 1.5 with adjusted p-value of less than 0.05.

Collection time	Sample Type	*Gene*	GO/KEGG pathway
June	WI-Fr	*NFD4*	Female gametophyte development
June	WI-Fr	*WRKY75*	Flower initiation
June	WI-Fr	*ERF1B*	Subset of AP2
June	WI-Fr	*RLP12*	Stamen
June	WI-Fr	*MYB108*	Stamen development
June	WI-Fr	*ERF113*	Flower initiation
June	WI-Fr	*MYB108*	Stamen development
June	WE-Fr	*YAB1/FIL1*	Meristem and organ identity
June	WE-Fr	*UFC*	Upstream of FLC
June	WE-Fr	*FD*	Promote flowering
June	WE-Fr	*UFO*	Floral meristem identity
June	WI-NFr	*RGL1*	Floral development
June	WI-NFr	*YAB4/INO*	Regulation of flower differentiation
June	WI-NFr	*EMS1*	Anther development
June	WE-NFr	*MADS6*	Early fruit development
June	WE-NFr	*CAL-A*	Transition to reproductive phase
June	WE-NFr	*VRN1*	Transition to reproductive phase
June	WE-NFr	*CAL-A/AP1*	Transition to reproductive phase
September	WE-Fr	*FLC/CAL-A*	
September	WE-Fr	*GASA14*	Plant growth
September	WI-Fr	*YAB4/INO*	Regulation of flower differentiation
September	WI-Fr	*RPK1*	Embryo development
September	WI-Fr	*RFK1*	Restoration of male sterility
September	WE-NFr	*CAL-A*	Transition to reproductive phase
September	WE-NFr	*DEF*	Flower morphogenesis
September	WE-NFr	*UFO*	Floral meristem identity
September	WE-NFr	*NAC056*	Seed morphogenesis
September	WI-NFr	*JMJ30*	Flower repressor
September	WI-NFr	*RGL1*	Floral development
September	WI-NFr	*CRY1*	Early flowering

There were 108 DEGs between ‘Wichita’ and ‘Western’ fruiting samples. Forty-nine of them were specific and unique to fruiting samples, 36 of these genes were upregulated in the ‘Wichita’ while the remainder were upregulated in ‘Western’ (Table 4). In September 2016, there were 127 DEGs between non-fruiting samples of ‘Wichita and ‘Western’. Forty-seven of these genes were specific to non-fruiting samples, out of which 29 genes were upregulated in the ‘Wichita’ non-fruiting samples and the remainder were upregulated in the ‘Western’ non-fruiting samples (18 genes) (Table 4). In September 2016, there were 196 DEGs between ‘Wichita’ and ‘Western’ fruiting samples. 116 of these genes were specific to the fruiting samples among which 60 were upregulated in the ‘Wichita’ samples and the remainder of the 56 genes were upregulated in the ‘Western’ fruiting samples.

### Flower bloom season

#### RNA-seq analyses of bud samples collected from ‘Wichita’ and ‘Western’ fruiting vs. non-fruiting during two sampling times in March

In the season prior to flowering, several genes were differentially expressed between our samples that may have role in the initiation of male and/or female flowers. After the end of the growing season and the subsequent harvest, pecan trees are in a dormancy state during late fall and winter. Microscopy images from dormant buds collected in February 2019, showed that catkin primordia were fully developed in ‘Western’ buds as opposed to the ‘Wichita’ buds (Fig 2). To further investigate the flower initiation/formation genetic signals, additional bud samples were collected from the ‘Western’ and ‘Wichita’ shoots in early March and late March 2018. On March 5, 2018, there were two DEGs (S5 Fig) between ‘Western’ non-fruiting and ‘Western’ fruiting samples. One gene was *PER7* (*Peroxidase 7*) which was upregulated in non-fruiting sample and the second gene was *MADS27* with higher expression in the ‘Western’ fruiting samples. Previous studies have reported higher expression of *MADS27* in the spur buds with a high flowering rate compared to other bud types in Apple (*Malus domestica* Borkh)[25].

There were 17 DEGs between the ‘Wichita’ non-fruiting samples and the ‘Wichita’ fruiting samples from samples collected on March 5^th^ (2018) (S5 Fig). Five of the genes were upregulated in non-fruiting samples and twelve were upregulated in fruiting samples (S6 Fig). *SPL7*, *FAR3*, *MS2* were upregulated in non-fruiting samples and *LTP1*, *CAL-A* and *MADS6* were upregulated in fruiting samples (Table 5).

**Table 5:** Selected DEGs between non-fruiting and fruiting sample (March 5 and March 22, 2018) from ‘Wichita’ (WI) and ‘Western’ (WE) cultivars. Second column indicates the sample in which the given gene was significantly higher expressed compared to the other fruiting (Fr)/non-fruiting (NFr) status within the same genotype. The cut-off for the DEGs were set to a minimum log2fold change of 1.5 with adjusted p-value of less than 0.05.

Collection time	Sample Type	Gene	GO/KEGG pathway
March 5th	WE-NFr	PER7	Response to environmental stresses
March 5th	WE-Fr	MADS27	flowering
March 5th	WI-NFr	SPL7	Control of flowering
March 5th	WI-NFr	FAR3	Flower organ development
March 5th	WI-NFr	MS2	Pollen development
March 5th	WI-Fr	LTP1	Flower development
March 5th	WI-Fr	CAL-A	Transition to reproductive phase
March 5th	WI-Fr	MADS6	Early fruit development
March 22nd	WI-NFr	18s rRNA	Repress YAB1, KNAT6, STM
March 22nd	WI-Fr	MYB 21/24	Male fertility
March 22nd	WE-NFr	organ-specific	P4-like protein
March 22nd	WI-Fr	auxin-induced protein	Pistillate development
March 22nd	WE-NFr	ABCG11	Cuticular lipids, pollen coat
March 22nd	WE-Fr	CAL-A	Transition to reproductive phase
March 22nd	WE-Fr	ARG2	Hormone signaling and fruit ripening
March 5th	WI-Fr	YAB4	Flower differentiation
March 5th	WI-Fr	BHLH93	Promote flowering
March 5th	WI-Fr	MYB108	Stamen development
March 5th	WI-Fr	ABCG11	Seed development
March 5th	WI-Fr	YAB1/FIL1	Meristem and organ identity
March 5th	WI-Fr	NAC054	Ovule development
March 5th	WI-NFr	GAS1	Flower development
March 5th	WI-NFr	ATH1	Flower repressor
March 5th	WI-NFr	DCN1	Pollen development
March 5th	WE-Fr	ABI2	Repressor
March 5th	WE-Fr	PMADS2	Male development
March 5th	WE-Fr	MADS23	Repressor of FT
March 5th	WE-Fr	FD	Promote flowering
March 5th	WE-Fr	AGL9	Flower development
March 5th	WE-Fr	1-Sep	Early flowering
March 5th	WE-NFr	LFY	Flower initiation
March 5th	WE-NFr	AGL9	Flower development
March 22nd	WI-Fr	TT10	Seed coat pigmentation
March 22nd	WI-Fr	RLP12	Stamen
March 22nd	WI-NFr	VIL1	Promote flowering
March 22nd	WI-NFr	VIL2	Promote flowering
March 22nd	WI-NFr	BHLH93	Promote flowering
March 22nd	WI-NFr	BHLH61	Promote flowering
March 22nd	WI-NFr	WOX3	Promote flower induction
March 22nd	WI-NFr	APPR9	Activation of CO
March 22nd	WI-NFr	ERF113	Flower initiation
March 22nd	WI-NFr	AHL1	Ovule development
March 22nd	WI-NFr	WER	Regulates FT
March 22nd	WI-NFr	ARG2	Floral transition
March 22nd	WI-NFr	ANT	Flower development (ovule)
March 22nd	WI-NFr	VIN3	FLC repression
March 22nd	WI-NFr	YAB2	Floral organ identity/leaf development
March 22nd	WI-NFr	AGL19	Activates LFY and AP1
March 22nd	WI-NFr	YAB1/FIL1	Meristem and organ identity
March 22nd	WI-NFr	MYC2	Floral repressor
March 22nd	WI-NFr	SLN1	Floral repressor
March 22nd	WI-NFr	OFP13	Suppress cell elongation
March 22nd	WI-NFr	COL11	Stamen
March 22nd	WI-NFr	BHLH137	Male sterility
March 22nd	WI-NFr	MIK2	Pollen development
March 22nd	WI-NFr	SRS1	Flowering time/Stamen development
March 22nd	WI-NFr	RFK1	Restoration of male sterility
March 22nd	WE-Fr	MADS23	Repressor of FT
March 22nd	WE-Fr	PMADS2	Male development
March 22nd	WE-Fr	NAC056	Regulate embryogenesis
March 22nd	WE-Fr	UFO	Floral meristem identity
March 22nd	WE-Fr	AG	Flower development
March 22nd	WE-Fr	DEF	Flower morphogenesis
March 22nd	WE-Fr	FLC	Floral repressor
March 22nd	WE-NFr	SAUR32	Apical hook opening
March 22nd	WE-NFr	ABI2	Repressor
March 22nd	WE-NFr	MYB24	Stamen development
March 22nd	WE-NFr	MYB114	Red color pigmentation

Bud samples collected on March 22, 2018, had 5 DEGs between the ‘Wichita’ non-fruiting samples and the ‘Wichita’ fruiting samples (S5 Fig). The only gene that was upregulated in the ‘Wichita’ nonfruiting samples on March 22^nd^ was 18s rRNA. There were 4 upregulated genes in fruiting samples including 2 uncharacterized genes, *MYB21/24* and an auxin-induced protein 15A-like.

Bud samples collected on March 22^nd^ had 10 DEGs between ‘Western’ non-fruiting and ‘Western’ fruiting samples (S5 Fig). These genes included an *ORGAN*-*SPECIFIC PROTEIN P4*-*LIKE* and *ABCG11* that were higher expressed in the non-fruiting samples and the remainder exhibited higher expression in fruiting samples (Table 5).

There were 71 DEGs between the ‘Wichita’ non-fruiting samples and the ‘Western’ non-fruiting samples (S7A Fig). Twenty of these genes were specific to non-fruiting samples. There were 100 DEGs between ‘Wichita’ fruiting samples and ‘Western’ fruiting samples. Forty-nine of these genes were specific to fruiting samples out of which 32 genes were upregulated in the ‘Wichita’ fruiting samples and 18 genes were upregulated in the ‘Western’ fruiting samples (S7B Fig). It is important to point out that in the early spring; catkin primordia are already completely formed in ‘Western’ buds while ‘Wichita’ buds continue to undergo the process of anther development. The function of these specific DEGs between samples collected from ‘Wichita’ and ‘Western’ in March 2018 require further evaluated for their roles in anther development.

On March 22^,^ 2018, there were 162 DEGs between ‘Wichita’ non-fruiting and ‘Western’ non-fruiting samples (S7B Fig). From these 162 genes, 101 genes were specific to non-fruiting samples. There were 90 DEGs between fruiting samples of ‘Wichita’ and ‘Western’. From these, 29 genes were specific to fruiting samples (S7 Fig).

**Fig 4:**
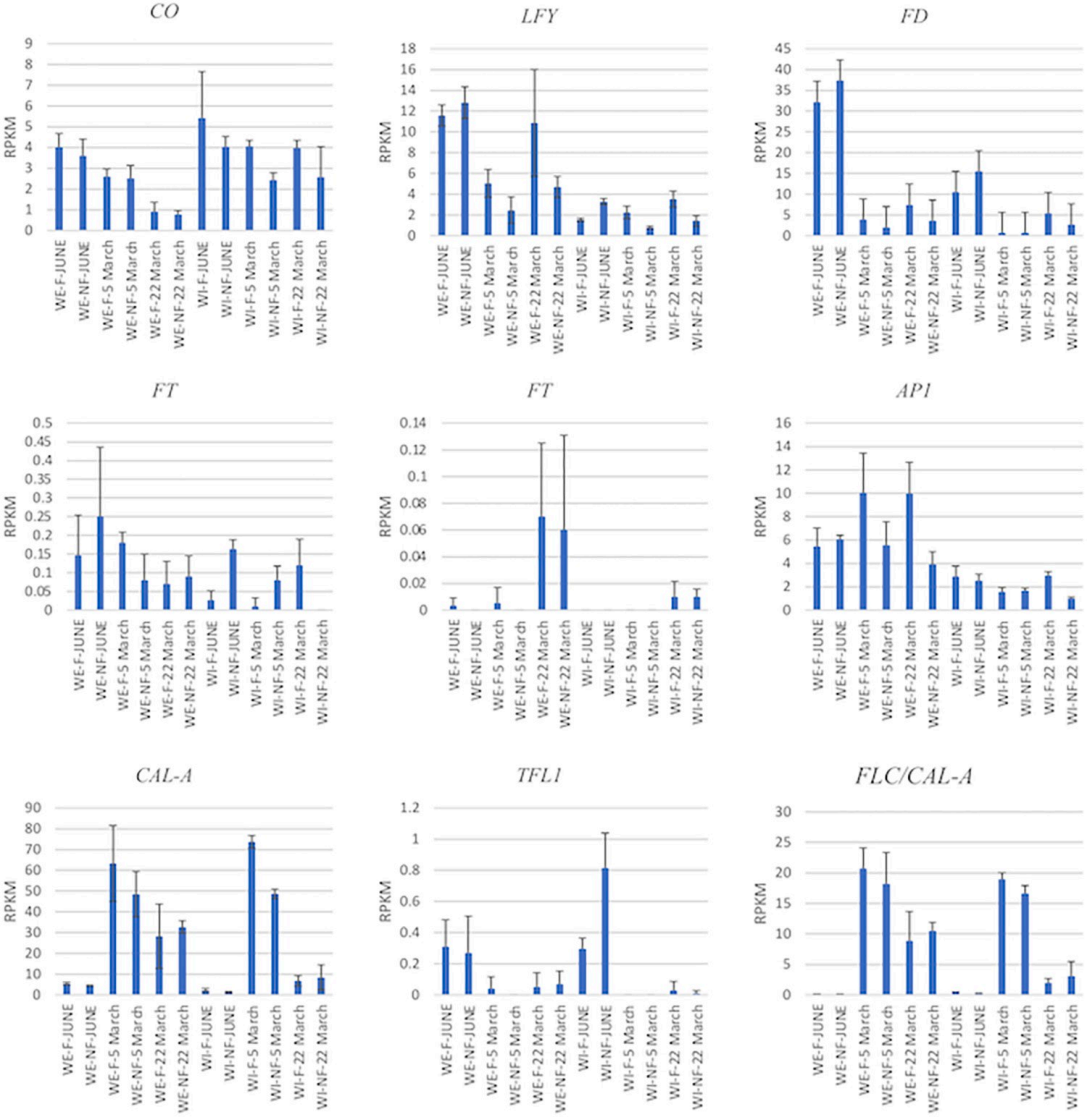
The expression of some of the canonical flowering genes in our RNA-Seq data. The RPKM of individual flowering genes is shown in regard to ‘Western’ (WE) and ‘Wichita’ (WI) throughout time on both fruiting (Fr) and non-fruiting (NFr) shoots. These samples were collected in June 2017 and March 2018. Error bars indicate standard error.

The expression of canonical flowering genes such as *CO*, *LFY*, *FD*, *FT*, *AP1*, *CAL-A*, *TFL1* and *FLC* were also detected in our samples (Fig 4).

### Quantitative RT-PCR

**Fig 5:**
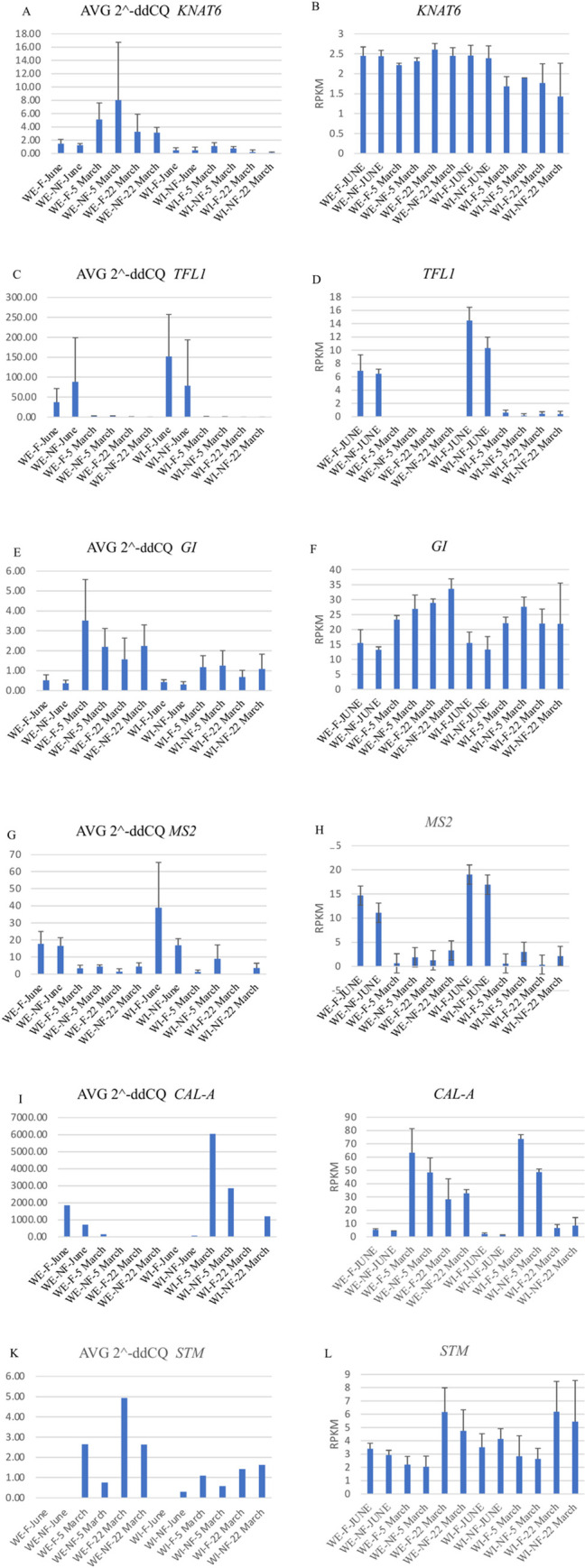
Validation of RNA-Seq through qPCR. Five genes were selected to verify RNA-Seq results using qRT-PCR. Graphs on the left (A, C, E, G, I, K) are 2^ddCQ values from qPCR and graphs on the right (B, D, F, H, J, L) show normalized gene expression (RPKM) values from RNA-Seq. Pearson correlations analyzes was performed to determine correlation between RNA-Seq normalized gene expression (RPKM) and qPCR (2^ddCQ).

To validate the accuracy and reproducibility of RNA-Seq analysis, six genes were randomly selected for qPCR analyses (Fig 5). Pearson correlation analyses was performed to find the correlation between the RNA-Seq normalized gene expression values (RPKM) and qPCR (2^ddCQ) values. For all the six genes, estimated correlation, R values were positive (S8 Fig). *TFL1* and *MS2* have significantly positive correlation between q-PCR and RNA-Seq with R = 0.960 and R = 0.914 respectively (S8B and S8D Fig).

## Discussion

In perennial plants such as *Carya illinoinensis*, once reproductive maturity is attained, there are genetic switches that are regulated and required for flower development year after year. To understand these genetic switches and their timing in pecan, this study focused on developing buds’ transcriptomes from two pecan cultivars, protandrous ‘Western’ and protogynous ‘Wichita’, the season before bloom and the season during bloom. ‘Western’ and ‘Wichita’ are widely grown in arid and semi-arid growing regions, such as the southwestern United States, where they are included in most commercial orchards. The formation of both male and female flowers on the same pecan tree has made defining what genes are specifically responsible for pistillate and catkin (or both) formation challenging at best. In previous studies, it was found that pecan tree shoots that had previously produced fruit were less likely to produce pistillate flowers the following season as opposed to those branches (shoots) that did not produce fruit [7, 8]. Our study showed that there were no statistical differences in catkin production on ‘Western’ trees as the shoots that had previously produced fruit had nearly the same number of catkins as the shoots that had not produced fruit (Fig 1). However, catkin production on ‘Wichita’ trees was profoundly different. There were statistically less catkins on ‘Wichita’ shoots that had pistillate flowers during the current season (Fig 1B). There were also statistically less catkins on the shoots that were not fruiting the previous season (Fig 1A). The microscopy analysis (Fig 2) of buds from ‘Western’ and ‘Wichita’ trees showed the definitive difference in timing for anther development. Due to these differences in timing between these cultivars, we surmised that it may be possible to distinguish the genetic signals and the timing for flowering initiation in the buds during development.

RNA-Seq analyses on the selected flowering genes from the bud samples collected from ‘Wichita’ and ‘Western’ cultivars revealed that the differences between the fruiting and non-fruiting shoots of ‘Wichita’ were more distinct than the fruiting and non-fruiting shoots of ‘Western’ in the season before bloom (June and September). Based on previous studies and microscopy images (Fig 2), we know catkin primordia formation begins in the dormant buds months in advance of bud break. However, the stage of catkin primordia development during the dormancy and budbreak periods differs between protogynous and protandrous genotypes. Therefore, it is reasonable to suggest that some of the flowering genes (*RLP12*, *MYB108*, *ERF113*, *NFD4*, *WRKY75*) that were differentially expressed between ‘Wichita’ and ‘Western’ samples (fruiting and non-fruiting) might have roles in catkin development (anther development) and could provide some preliminary clues for dichogamy in pecan. *MYB* genes have been shown to function in the formation of male flowers in other plant species. In other plant species, the *MYB* genes are involved in a transcriptional cascade that mediates stamen and pollen maturation [26–29]. The expression of these genes is mostly confined to sporophytic tissues of the stamen. The *myb108* mutant exhibited reduced male fertility and this phenotype was associated with reduced pollen viability and delayed anther dehiscence [19]. Previous studies have shown that *MYB24* and *MYB108* have overlapping functions that facilitates stamen and pollen maturation in response to jasmonic acid (JA) [19]. In Arabidopsis, jasmonate is an essential key signal required for stamen and pollen maturation which leads to male fertility [30]. Previous studies in Arabidopsis suggested that *MYB21* and *MYB24* specifically regulate male fertility and the *MYB21/24* double mutant exhibited male sterility due to defects in anther dehiscence, pollen maturation and filament elongation [26]. *MYB21* and *MYB24* along with another member of *MYB* family, *MYB57*, are GA-dependent stamen-enriched genes and the triple mutants in Arabidopsis conferred short stamen and lead to male sterility [27]. Other studies have shown that *MYB21* and *MYB24* promote petal and stamen development and have a role in gynoecium growth [28]. The auxin-induced protein 15A-like belongs to the *ARG7* gene family. The *ARG7* gene family can regulate plant growth hormones and affect pistil development [31]. Previous studies on pecan pistillate flower transcriptome also showed that *ARG7* played a role in pecan floral organ development [9].

The catkin bloom patterns revealed that pistillate formation on ‘Wichita’ cultivar shoots negatively impacted catkin production on the same shoot in the same year. As ‘Western’ does not follow the same pattern, it is likely that the differentially expressed genes that were upregulated in the ‘Western’ samples are most likely related to catkin initiation/formation. Some DEGs with higher expression in the ‘Western’ fruiting shoots collected in June were most likely related to catkin initiation and genes highly expressed in the ‘Western’ fruiting samples from September could be putative genes for anther development. As it is expected for ‘Wichita’ catkin primordia to fully develop right before bloom, it is compelling that some higher expressed genes (*MYB108*, *DCN1*, *RLP12*, *COL11*, *BHLH137*, *MIK2*, *RFK1*, *SRS1*) in ‘Wichita’ samples compared to ‘Western’ samples in March were putative candidate genes for anther and pollen development in pecan. Comparison of ‘Wichita’ fruiting and non-fruiting samples also suggested *SPL7* and *MS2* as two other possible catkin specific genes. The *SPL7* gene (*SQUAMOSA PROMOTER BINDING-LIKE7)* is one of the miR156-targeted *SPL* genes that is involved in the control of phase transition and flowering by regulating *AP1* and *FUL* in Arabidopsis [32–34]. miR156 and its target, the *SPL* gene, control the aging pathway of flowering [35]. The aging pathway ensures flowering occurs, even under noninductive conditions [32, 36–38]. Overexpression of *SPL7* in switchgrass promoted flowering whereas downregulation of this gene moderately delayed flowering [39]. The *MALE STERILITY 2 (MS2)* gene encodes a protein that is involved in male gametogenesis and is required for proper pollen development [15].

Shoots that did not bear fruit in 2017 for both ‘Wichita’ and ‘Western’ were most likely to bear pistillate flowers in May 2018. Our data showed a considerable number of DEGs between non-fruiting shoots of ‘Wichita’ and ‘Western’ in March 2018. Higher expression of some flower repressor genes (*MYC2*, *SLN1*) and *BHLH137* (male sterility) in ‘Wichita’ non-fruiting samples were observed on March 22, 2018. Higher expression of these genes could potentially repress catkin development and bloom in ‘Wichita’ trees. Unfortunately, due to the destructive nature of sample collection for RNA-Seq, we lack the information regarding the ultimate fate of these buds.

## Conclusions

To the best of our knowledge, no significant differences in the number of catkins between ‘Western’ and ‘Wichita’ have previously been reported. Here we show that there is a negative impact on staminate bloom on shoots that currently have pistillate flowers, suggesting that pistillate flower production in ‘Wichita’ may occur at the expense of catkin production but no differences were observed for ‘Western’ catkins. This study needs to be performed on additional protandrous and protogynous pecan cultivars to determine if there is indeed a direct relationship between pistillate flower and catkin production on protogynous pecan cultivars. Based on the previous studies, it was hypothesized that in pecan, flower initiation for both staminate and pistillate flowers occur one season before bloom. Our data presented gene expression evidence for the initiation of both flower types in the year before bloom, however, since both ‘Wichita’ and ‘Western’ non-fruiting shoots were expected to bear fruit the following year, and the caveat of alternate bearing is proportional (flowering shoots are **less likely** to produce fruit the following year), defining the pistillate specific genes were especially challenging. The current study specifically looked at the differentially expressed genes. Our previous analyses presented the expression of hundreds of known flowering genes, among which, several were known to be female specific genes in other species [10]. Further studies with more time points of sample collection are required for more comprehensive insight into the timing of pecan pistillate specific genes expression and a better understanding of the function of each gene in pecan.

**Fig 6:**
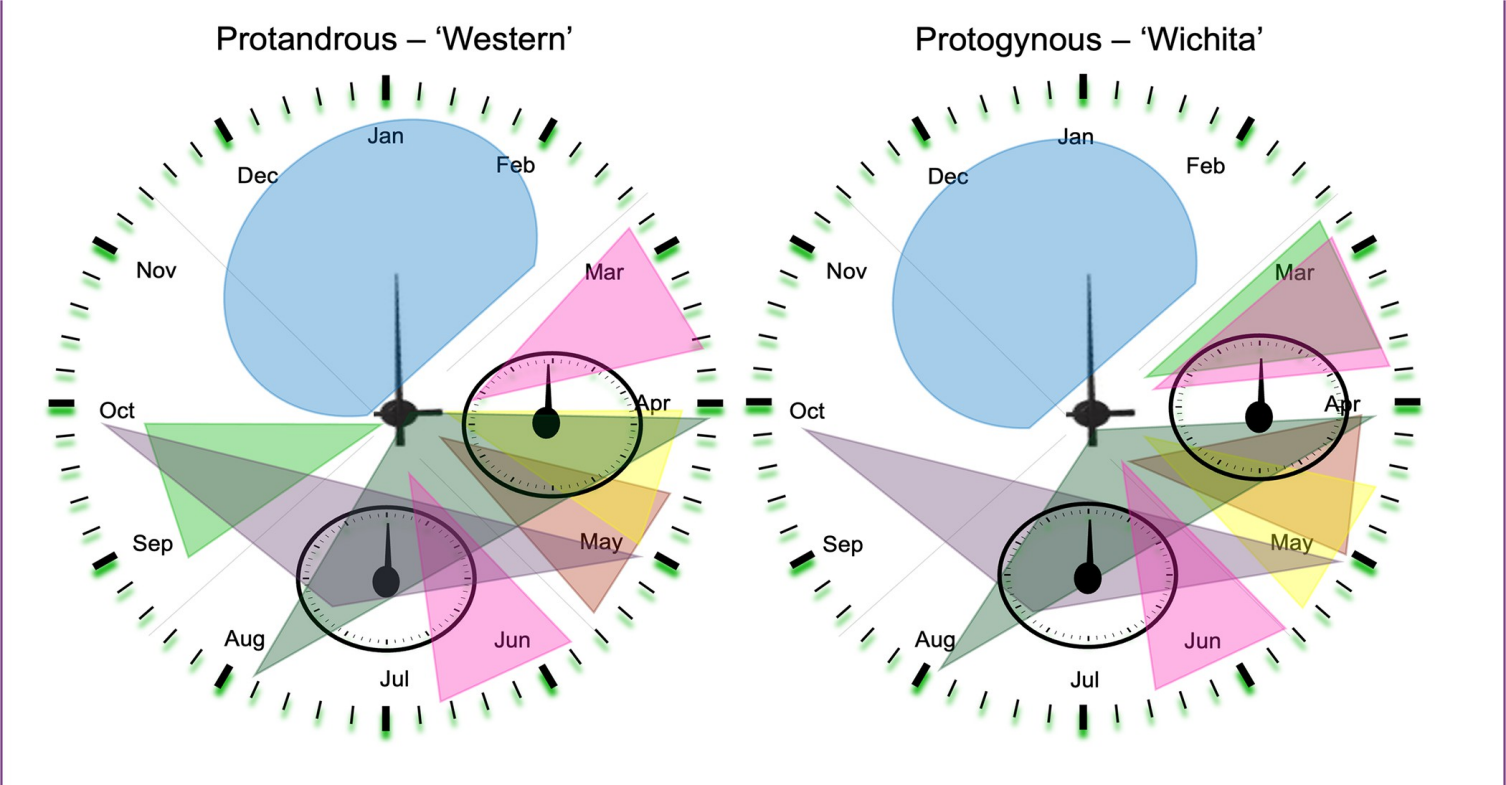
Schematic of the proposed flowering “clocks” in North American grown pecan.

Our data indicates that pecan flowering is evidently programmed to follow several precise ‘clocks’ (Fig 6) and the elements of these clocks will be used as genetic resources and background for better understanding of the flower initiation mechanisms in pecan. Further comprehensive studies of the selected candidate genes may enable researchers to understand the timing of floral initiation and ultimately assist in mitigation of alternate bearing effects and help the breeders to control or select specific traits of flowering in pecan.

Both protandrous and protogynous cultivars are dormant during the winter months. In spring, signals responsible for the production of female flowers occurs for both protogynous and protandrous trees. Protogynous trees begin anther development in their buds in early spring. Protandrous trees already have fully developed anthers in early spring. In late April and early May both protogynous and protandrous pecan trees have pistillate flower bloom and pollination and fertilization occur. After successful pollination, fruits develop throughout the summer. In a separate internal clock, flower signals are active in the bud for the next year and catkin primordia are forming in the bud. At the end of the summer, protandrous cultivars go through the development of anther primordia in the bud for the next year while no further development occurs in protogynous cultivars. Trees go dormant in winter and this cycle continues.

## Supporting information

S1 Fig Venn diagrams of the DEGs between samples collected in June and September 2018. This figure indicated a higher number of differentially expressed genes between the fruiting and non-fruiting samples from the ‘Wichita’ cultivar compared to the fruiting and non- fruiting samples from the ‘Western’ cultivar.
(TIF)

S2 Fig Log2 fold changes for the selected DEGs between ‘Wichita’ non- fruiting and fruiting samples in June 2018. (TIF)

S3 Fig Log2 fold changes for the selected DEGs between ‘Wichita’ non- fruiting and fruiting samples in September 2018. (TIF)

S4 Fig Venn diagrams of DEGs between ‘Wichita’ and ‘Western’ fruiting and non-fruiting samples in June and September. (TIF)

S5 Fig Venn diagrams of DEGs between ‘Wichita’ and ‘Western’ fruiting and non-fruiting samples in March. (TIF)

S6 Fig DEGs between the ‘Wichita’ non-fruiting fruiting samples collected on March 5^th^. (TIF)

S7 Fig DEGs between the ‘Wichita’ and ‘Western’ fruiting and non-fruiting samples collected on March 22^nd^. (TIF)

S8 Fig Pearson correlation analyses between gene expression from RNA-Seq and qPCR to validate RNA-Seq data. (TIF)

S1 Table Details on catkin counts from ‘Wichita’ and ‘Western’ trees in 2019. (XLSX)
