# Supplementary figures and images for: Comparative transcriptome analyses reveal insights into catkin bloom patterns in pecan protogynous and protandrous cultivars
Source: PLoS One. 2023 Feb 16;18(2):e0281805. doi: 10.1371/journal.pone.0281805 (PMC9934368; doi:10.1371/journal.pone.0281805)

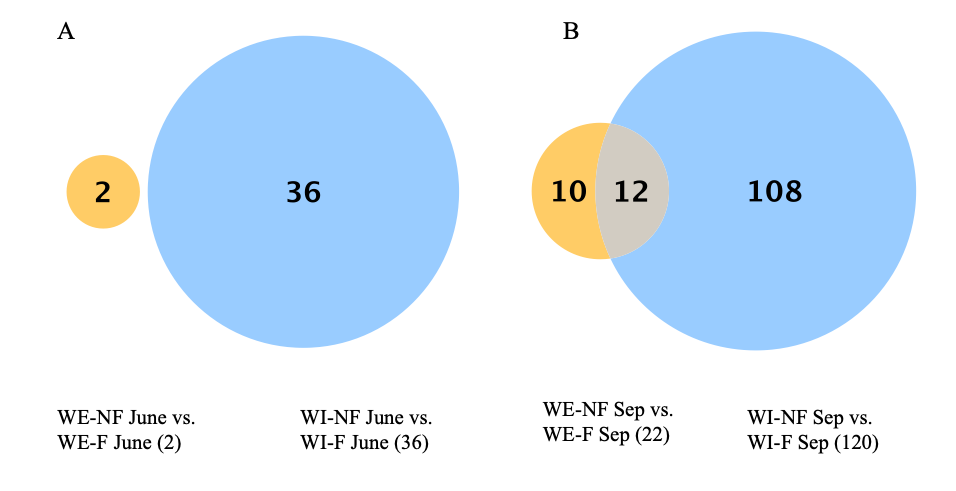

Supplement: S1 Fig — This figure indicated a higher number of differentially expressed genes between the fruiting and non-fruiting samples from the ‘Wichita’ cultivar compared to the fruiting and non- fruiting samples from the ‘Western’ cultivar. (TIF) [file pone.0281805.s001.tif]

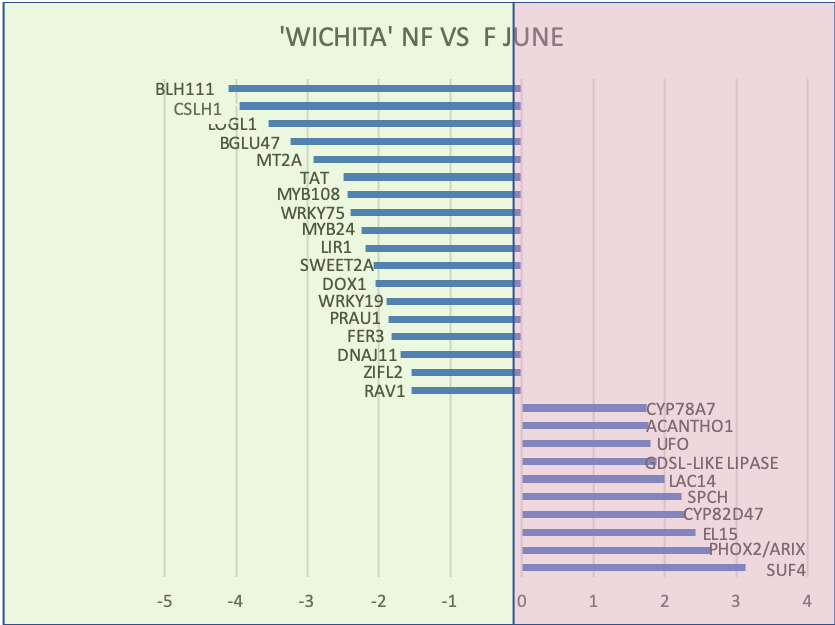

Supplement: S2 Fig — (TIF) [file pone.0281805.s002.tif]

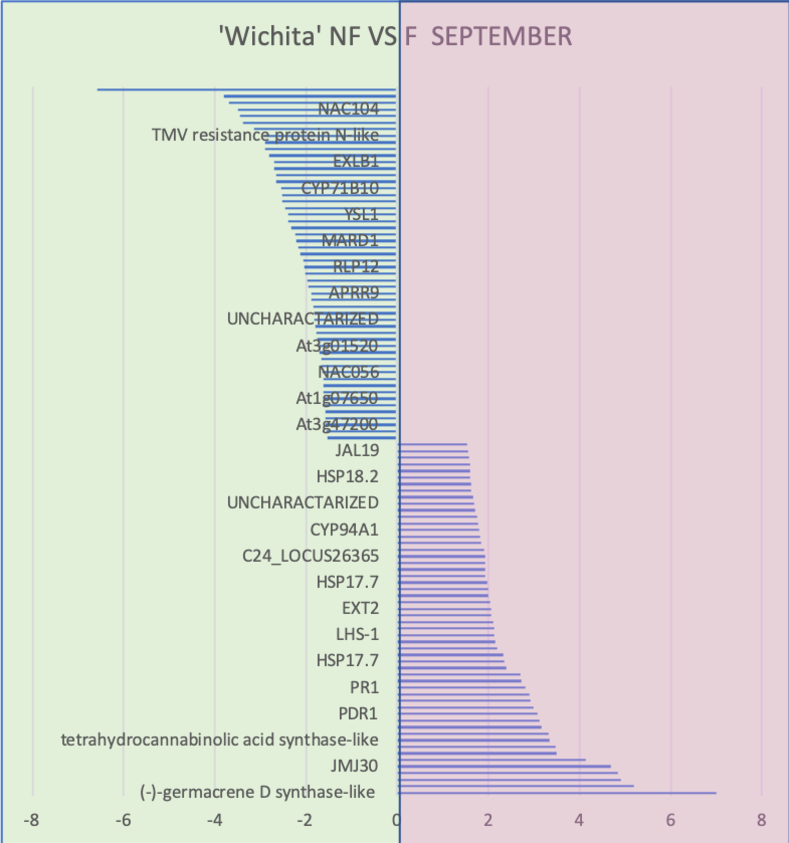

Supplement: S3 Fig — (TIF) [file pone.0281805.s003.tif]

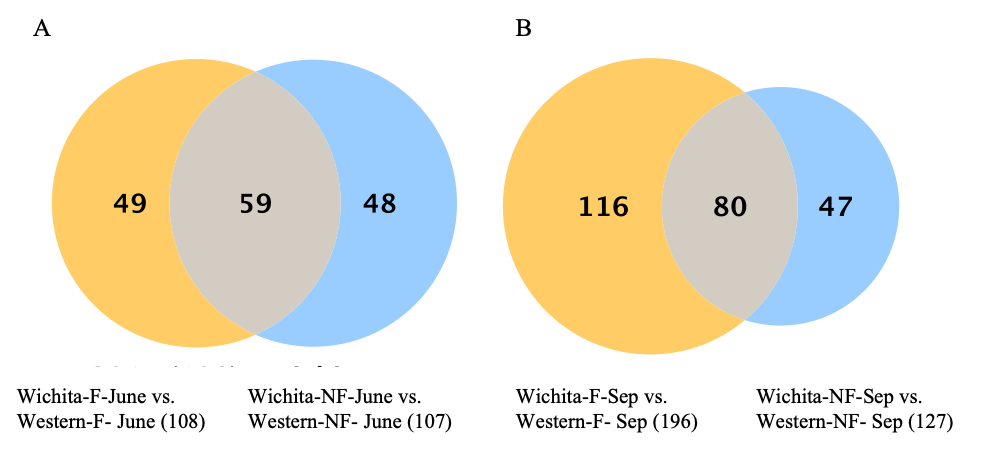

Supplement: S4 Fig — (TIF) [file pone.0281805.s004.tif]

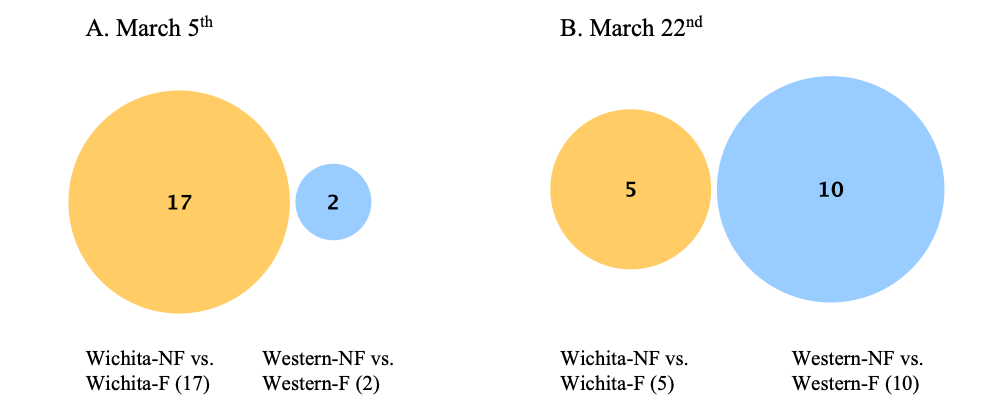

Supplement: S5 Fig — (TIF) [file pone.0281805.s005.tif]

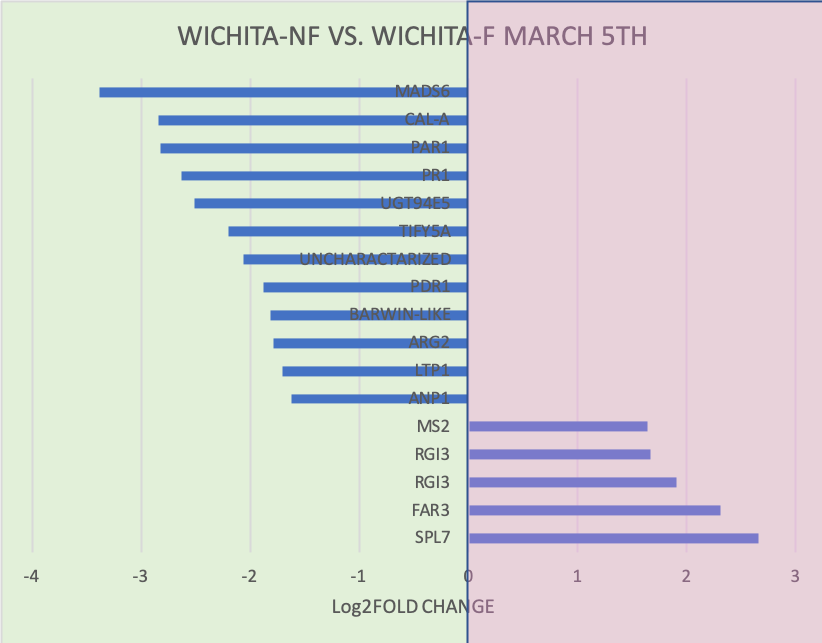

Supplement: S6 Fig — (TIF) [file pone.0281805.s006.tif]

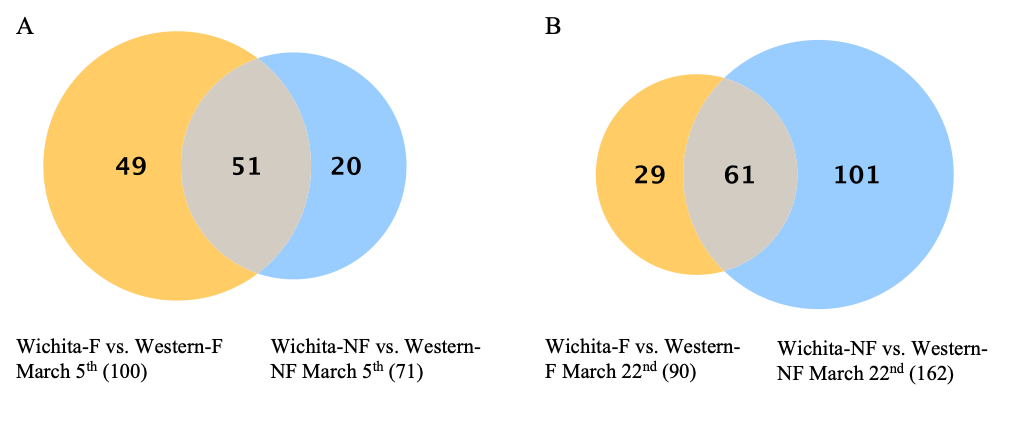

Supplement: S7 Fig — (TIF) [file pone.0281805.s007.tif]

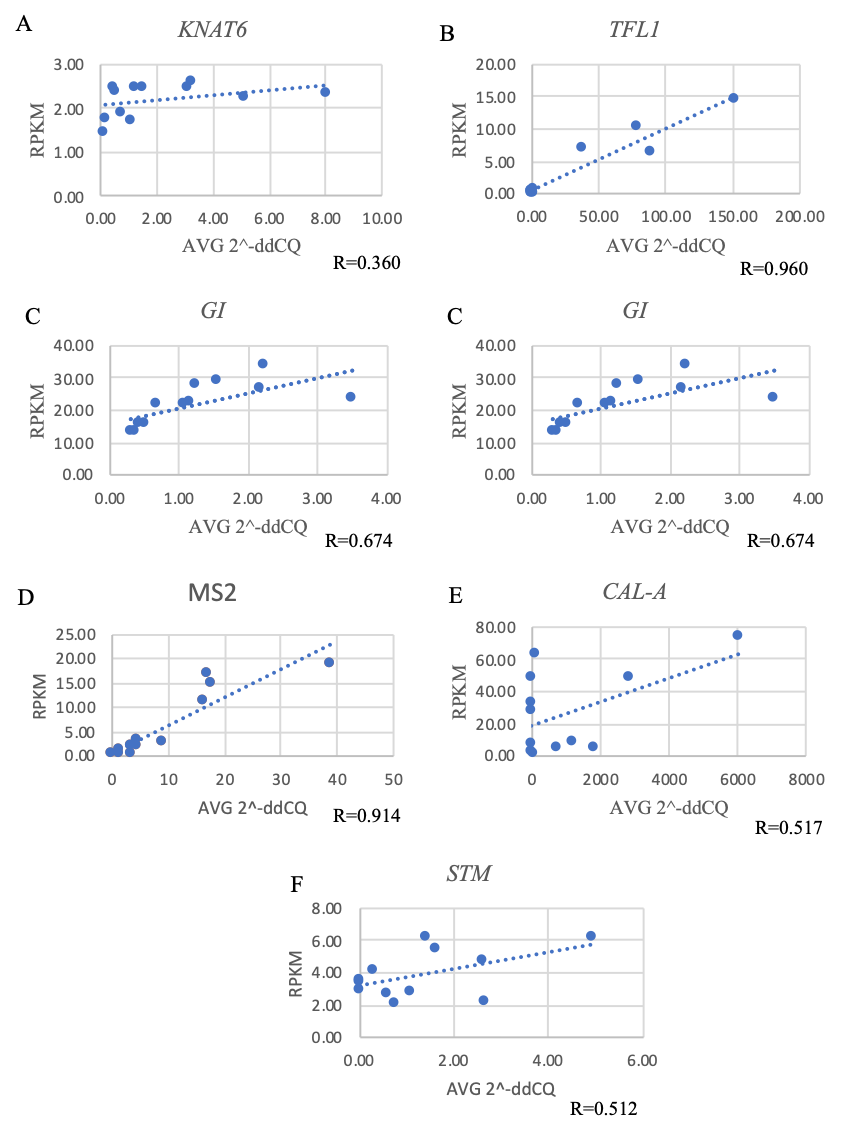

Supplement: S8 Fig — (TIF) [file pone.0281805.s008.tif]
